# Circulating biomarkers of antioxidant status and oxidative stress in people with cystic fibrosis: A systematic review and meta-analysis

**DOI:** 10.1016/j.redox.2020.101436

**Published:** 2020-01-23

**Authors:** Adam J. Causer, Janis K. Shute, Michael H. Cummings, Anthony I. Shepherd, Mathieu Gruet, Joseph T. Costello, Stephen Bailey, Martin Lindley, Clare Pearson, Gary Connett, Mark I. Allenby, Mary P. Carroll, Thomas Daniels, Zoe L. Saynor

**Affiliations:** a School of Sport, Health and Exercise Science, Faculty of Science and Health, University of Portsmouth, Portsmouth, UK; b Cystic Fibrosis Unit, University Hospital Southampton NHS Foundation Trust, Southampton, UK; c School of Pharmacy and Biomedical Sciences, Faculty of Science and Health, University of Portsmouth, Portsmouth, UK; d Department of Diabetes and Endocrinology, Queen Alexandra Hospital, Portsmouth, UK; e Laboratory of Impact of Physical Activity on Health (IAPS), UR n°201723207F, University of Toulon, France; f School of Sport, Exercise and Health Sciences, Loughborough University, Loughborough, UK; g National Institute for Health Research, Southampton Biomedical Research Centre, Southampton Children's Hospital, Southampton, UK

**Keywords:** Biological markers, Glutathione, Minerals, Oxidoreductases, Redox imbalance, Respiratory disease, Vitamins

## Abstract

**Introduction:**

Oxidative stress may play an important role in the pathophysiology of cystic fibrosis (CF). This review aimed to quantify CF-related redox imbalances.

**Methods:**

Systematic searches of the Medline, CINAHL, CENTRAL and PsycINFO databases were conducted. Mean content of blood biomarkers from people with clinically-stable CF and non-CF controls were used to calculate the standardized mean difference (SMD) and 95% confidence intervals (95% CI).

**Results:**

Forty-nine studies were eligible for this review including a total of 1792 people with CF and 1675 controls. Meta-analysis revealed that protein carbonyls (SMD: 1.13, 95% CI: 0.48 to 1.77), total F_2_-isoprostane 8-iso-prostaglandin F_2α_ (SMD: 0.64, 95% CI: 0.23 to 1.05) and malondialdehyde (SMD: 1.34, 95% CI: 0.30 to 2.39) were significantly higher, and vitamins A (SMD: −0.66, 95% CI -1.14 to −0.17) and E (SMD: −0.74, 95% CI: −1.28 to −0.20), β-carotene (SMD: −1.80, 95% CI: −2.92 to −0.67), lutein (SMD: −1.52, 95% CI: −1.83 to −1.20) and albumin (SMD: −0.98, 95% CI: −1.68 to −0.27) were significantly lower in the plasma or serum of people with CF versus controls.

**Conclusions:**

This systematic review and meta-analysis found good evidence for reduced antioxidant capacity and elevated oxidative stress in people with clinically-stable CF.

## Highlights

• Blood biomarkers of oxidative stress were elevated in stable CF vs non-CF controls.
• Lipid peroxidation was positively correlated with age and immune cell count in CF.
• Antioxidants vitamins A & E, β-carotene, lutein and albumin were lower in stable CF.
• Antioxidants were positively correlated with body mass index and lung function in CF.

## Graphical abstract

**Figure:**
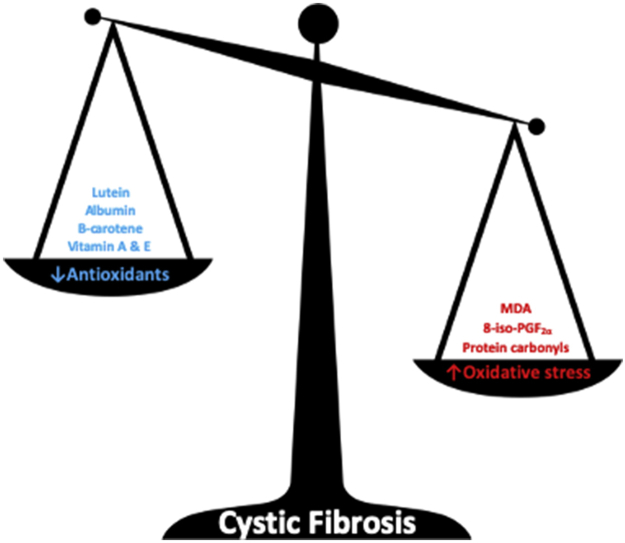

## 1 Introduction

Cystic fibrosis (CF) is a multi-system, life-shortening, autosomal recessive disease affecting more than 70,000 people globally. The disease occurs as a result of mutations in the gene encoding for the CF transmembrane conductance regulator (CFTR), the primary function of which is the efflux of chloride and bicarbonate anions. CFTR is commonly expressed within the epithelial cells that line the mucous membrane and submucosal glands of the airway [1]. Dysfunctional CFTR results in inflammation [2] and airway infection, a progressive decline in lung function [3] leading to respiratory failure and premature death [4]. CFTR is also expressed within various other bodily systems, including the gastrointestinal tract, pancreas, sweat ducts, skeletal muscle, cardiovascular and reproductive organs [5]. CF is, therefore, a complex multisystem condition requiring repeated comprehensive assessments to monitor for and treat disease complications.

Transient increases in free radicals derived from oxygen (reactive oxygen species) and nitrogen (reactive nitrogen species) activate various physiological signalling cascades that are beneficial for cellular function and communication [6]. However, several CF co-morbidities, including lung disease [7], inflammation [8], systemic hypoxaemia [9,10] and dysglycaemia [11] lead to the exacerbated production of reactive oxygen species exceeding the levels required for optimal physiological function. CFTR has also been implicated in the efflux of the main non-enzymatic antioxidant, reduced glutathione (GSH) [12]. Therefore, a loss of CFTR function *in vitro* leads to an increased accumulation of intracellular GSH, which blunts the response to redox-sensitive signalling pathways that modulate adaptations to hypoxia [13] and cigarette smoke [14], and may render extracellular compartments more susceptible to oxidative stress [15]. In addition to this, polymorphisms of the glutathione S-transferase (GST) and the glutamate-cysteine ligase genes are likely to further deplete extracellular synthesis and the detoxification potential of GSH [12,16,17]. Together, these factors in addition to the malabsorption of micronutrients with antioxidant properties in CF [18], causes an imbalance between oxidants and antioxidants in favour of oxidants (i.e. oxidative stress), the disruption of redox signalling and molecular damage [6].

Whilst the study of CF-related redox imbalances dates back as far as 1967 [19] and has been the topic of several narrative reviews [15,20,21], a recent meta-analysis highlighted the potential significance of these imbalances when considering disease pathophysiology [22]. Van t’ Erve et al. [22] observed that a biomarker of lipid peroxidation, F_2_-isoprostane 8-iso-prostaglandin F_2α_ (8-iso-PGF_2α_), was higher in CF compared to other diseases, including lifestyle factors and environmental exposures which are conventionally characterised by oxidative stress, such as tobacco smoking and cardiovascular disease [22]. Whilst this review identified some evidence for increased oxidative stress in CF, the range of biomarkers considered was limited [23].

Recent advances in analytical methodologies mean that previously inaccessible biomarkers, including those used to quantify oxidative damage in biological samples, are now more widely available for medical and physiological research purposes [24]. Similarly to elevated 8-iso-PGF_2α_ and malondialdehyde (MDA) being indicative of lipid peroxidation, oxidative modifications to proteins and DNA can be investigated by quantifying protein carbonyl groups and 8-hydroxy-20-deoxyguanosine (8-OHdG), respectively [25]. In addition to investigating the end-product of oxidative damage, it is also now possible to characterise antioxidant deficiencies that might be suitable therapeutic targets. It is recommended that vitamin and trace element concentrations are monitored in CF clinics [26]; however, from an antioxidant-perspective it is also useful to quantify thiols, such as GSH and cysteine (Cys), as these molecules are essential to understanding the redox potential (Eh) of biological fluids [6]. The content and activity of oxidoreductases, such as superoxide dismutase (SOD), catalase (CAT) and glutathione peroxidase (GPx), provide additional functional information regarding the maintenance of thiol:disulfide couples and are promising therapeutic targets [27]. Despite the array of available biomarkers to study redox imbalances, there is no systematic evidence to suggest that oxidative stress occurs as a consequence of lowered antioxidants in the blood.

Therefore, a systematic search of the existing evidence base from investigations of CF-related redox imbalances is needed to provide comprehensive and contemporary recommendations for subsequent clinical practice and research trials. This review aimed to summarise the literature investigating whether redox abnormalities are present in the blood of people with clinically-stable CF. We hypothesised that: *(1)* blood markers of antioxidant status would be lowered in people with clinically-stable CF compared to non-CF controls, and *(2)* blood markers of oxidative stress would be elevated in people with clinically-stable CF compared to non-CF controls.

## 2 Methods

### 2.1 Protocol registration

The review protocol was registered on PROSPERO (Reference: CRD42018094241; https://www.crd.york.ac.uk/prospero/display_record.php?ID=CRD42018094241).

### 2.2 Eligibility criteria

Included studies reported blood biomarkers of oxidative stress and/or antioxidant status in humans with CF, and were compared to a non-CF control group. We included population-based studies with a case-control comparison and intervention trials which had a case-control comparison at baseline. A clinical diagnosis of CF must have been supported by an abnormal sweat test, diagnostic genotyping and/or the participant being recruited through a CF clinic. Study participants must have been classed as clinically-stable and free from symptoms of an acute respiratory exacerbation at the time of testing, as this is a commonly accepted source of antioxidant deficiency and oxidative stress [28]. Studies were excluded if they were review articles, case reports or letters to the editor. Studies which compared results from individuals with CF versus standard values derived from the literature were excluded. There was no restriction on the type of redox-related biomarker; however, it must have been analysed in blood as CF is associated with renal disease [29] and pelvic floor incontinence [30], both of which may mean local inflammation alters the redox state of urine. There were no restrictions on publication period. Studies were excluded if the full-text was not available in English or French.

### 2.3 Information sources

Extensive searches of the Medline, Current Nursing and Allied Health Literature (CINAHL), Cochrane Central Register of Controlled Trials (CENTRAL) and PsycINFO databases were conducted to identify eligible studies up to October 9, 2019.

### 2.4 Searches

In accordance with similar systematic reviews in this area [31], the current review utilised a three-part search process to identify studies matching the eligibility criteria. The first strategy used Medical Subject Heading (MeSH) or subject terms, as follows: “cystic fibrosis” [MeSH] AND (antioxidants [MeSH] OR oxidoreductase [MeSH] OR vitamins [Pharmacological Action] OR oxidants [MeSH] OR “oxidative stress” [MeSH] OR ″ lipid peroxidation” [MeSH]) AND blood [subheading]. When compared to the eligibility criteria, 11 independent and eligible studies provided a list of 16 common biomarkers in plasma, serum, erythrocyte or whole blood samples. These data were then utilised to inform the second phase of the literature search.

The second strategy identified references based on specific words found within the title and/or abstract (ti/ab). In this search, the biomarkers and biological substances identified in the MeSH search were AND-linked to “cystic fibrosis”, as follows: “cystic fibrosis” [tiab] AND (tocopherol [tiab] OR carotene [tiab] OR malondialdehyde [tiab] OR MDA [tiab] OR “thiobarbituric acid reactive substances” [tiab] OR TBARS [tiab] OR ascorb*[tiab] OR lycopene [tiab] OR “protein carbolyl” [tiab] OR copper [tiab] OR isoprostane [tiab] OR prostaglandin [tiab] OR glutathione [tiab] OR hydroperoxide [tiab] OR superoxide [tiab] OR SOD [tiab] OR vitamin [tiab] OR zinc [tiab]) AND (blood [tiab] OR plasma [tiab] OR serum [tiab] OR erythrocyte [tiab] OR “whole blood” [tiab]). The MeSH search was also repeated at this stage to ensure a broad, specific and sensitive search [32].

The full search terms and strategies of the present review have been published on PROSPERO (Reference: CRD42018094241). Additionally, bibliographies of included studies and known review articles, clinical trial registers and conference proceedings were hand-searched by authors. Experts in the field (identified by the lead authors in published studies) were contacted to find unpublished trials.

### 2.5 Study selection

The screening and selection process followed two steps and was completed independently by two authors (AJC and ZLS). After removing duplicate articles, the ti/ab of all references were screened to identify whether the study met the eligibility criteria, with exclusion of ineligible articles. Full-texts of the remaining references were subsequently retrieved and compared with eligibility criteria. All eligible references were included in the systematic review (Table 1). Meta-analyses were only conducted on outcomes which were reported by a minimum of two independent studies. If a biomarker was the subject of two references by the same laboratory, only the most recent reference was included for meta-analysis. These methodologies have been used in similar meta-analyses [31,33].

**Table 1:** Characteristics of included studies.

First author [reference], study design	Location	Cases; n and characteristics	Controls; n and characteristics	Biomarker of interest
AbdulWahab [69], cross-sectional	Doha, Qatar	53 people with cystic fibrosis (CF) (M = 41.5%; 15.1 ± 9.1 y) from a kindred Arab tribe. 84.9% were I1234V homozygous and pancreatic sufficient (PS); 15.1% were non I1234V and pancreatic insufficient (PI); 35.8% had chronic *P. aeruginosa* colonisation; Mean ± SD forced expiratory volume in 1 s (FEV_1_) was 79.1 ± 22.2%. All those with PI were administered with pancreatic enzyme replacement and multivitamin supplements. 17.7% who were PS were receiving multivitamins.	45 healthy control participants (HCP) (male (M) = not reported (NR); 20.4 ± 10.1 y) were recruited from the same kindred Arab tribe.	Plasma albumin (CF group only) and zinc.
Olveira [58], cross-sectional	Malaga, Spain	36 people with CF (M = 56.3%; 27.2 ± 8.9 y). In those with frequent exacerbations, bacterial colonisation and/or FEV_1_ ≤ 50% predicted, 500 mg/day of azithromycin was administered (63.9%). Of those administered with azithromycin, mean ± SD FEV_1_ was 47.1 ± 21.3% and forced vital capacity (FVC) was 60.1 ± 21.8%; 69.6% were PI; 30.4% had CF-related diabetes (CFRD). Of those who were not administered with azithromycin, mean ± SD FEV_1_ was 77.1 ± 20.9% and FVC was 82.9 ± 17.1%; 61.5% were PI; 38.5% had CFRD. None were receiving dietary supplements with Omega-3 fatty acids.	41 HCP (M = 41.5%; 29.0 ± 9.6) matched for nutritional status, sex and age.	Plasma glutathione peroxidase (GPx), total antioxidant capacity (TAC), catalase (CAT) and superoxide dismutase (SOD). Plasma or serum F_2_-isoprostane 8-iso-prostaglandin F_2α_ (8-iso-PG2α), Thiobarbituric acid reactive substances (TBARS), vitamins A, D and E, and zinc.
Konstantinidis [139], cross-sectional	Thessaloniki, Greece	58 people with CF; 27 with (CF_w_NP) (M = 48.1%; 28.5 ± 6.1 y) and 31 without (CF_s_NP) (M = 54.8%; 28.1 ± 6.5 y) nasal polyposis; 25.9% were F508del homozygous; 50.0% were F508del heterozygous; 24.1% were non-F508del. 32.8% had *P. aeruginosa* colonisation. All CF participants were supplemented with 3000 IU/day of Vitamin D3.	62 people with chronic rhinosinusitis; 32 with (CRS_w_NP) (M = 53.1%; 29.3 ± 8.4 y) and 30 without (CRS_s_NP) (M = 46.7%; 29.5 ± 5.9 y) nasal polyposis. 32 HCP (M = 53.0%; 28.2 ± 7.9 y) from an outpatient clinic for elective nasal or oral surgery.	Serum 25(OH) VD3.
Antus [74], cross-sectional	Budapest, Hungary	40 people with CF (M = 60.0%; 25.0 ± 0.9 y); 50% were F508del homozygous. Mean ± SD FEV_1_ was 54.4 ± 4.0% and FVC was 73.5 ± 3.4%; 52.5% had *P. aeruginosa* colonisation. All were taking vitamin supplements, pancreatic enzyme supplements, inhaled/nebulised β_2_-agonists. 31.1% were receiving inhaled corticosteroid and 75.6% were receiving nebulised DNase. 31.1% were receiving inhaled antibiotics at the time of sample collection.	25 HCP (M = 52.0%; 35.8 ± 2.4 y) with no evidence of pulmonary disease.	Plasma malondialdehyde (MDA).
Lee [45], cross-sectional	Atlanta, USA	25 people with CF (M = 56.0%; 18.5 ± 14.0 y); 88% were PI; 48.0% were administered with vitamin D supplements.	28 HCP (M = 21.0%; 29.0 ± 6.0 y) on no medications which affect vitamin D concentration and metabolism. Vitamin D intake was limited to <1000 IU/day.	Serum albumin, total and free 25(OH)D and vitamin D binding protein.
Turowski [140], flaxseed supplement non-randomized and non-controlled trial	Philadelphia and Pennsylvania, USA	10 people with CF (M = 30.0%; 31.9 ± 10.8 y; FEV_1_ 76.8 ± 16.8% predicted; body mass index (BMI) 22.0 ± 1.8 kg/m^2^); 70.0% were PI, 80.0% taking pancrealipase.	5 HCP (comparable in age).	Plasma enterodiol and enterolactone.
Yadav [59], cross-sectional	Chandigarh, India	21 people with CF (M = 81.5%; 5.7 ± 2.5 y; this includes those with an acute respiratory exacerbation); 22.2% were receiving pancreatic enzyme supplementation.	27 HCP (M = 70.4%; 7.4 ± 2.7 y) who were matched for age and sex.	Plasma 25(OH)D, and vitamins A and E. Serum copper, iron and zinc.
Sadowska-Bartosz [53], cross-sectional	Rzeszow, Poland	22 people with CF; 12 with chronic *P. aeruginosa* (M = 30.0%; 12.8 ± 7.6 y; BMI 18.7 ± 2.9 kg/m^2^) and 10 with chronic *S. aureus* (M = 30.0%; 10.2 ± 3.6 y; BMI 18.6 ± 3.8 kg/m^2^) infections; 40.1% for F508del homozygous. Of those with *P. aeruginosa* colonisation, mean ± SD FEV_1_ was 71.0 ± 8.7% predicted and FEV_1_/FVC ratio was 79.7 ± 20.0%. Of those with *S. aureus* colonisation, mean ± SD FEV_1_ was 94.2 ± 11.4%, and FEV_1_/FVC ratio was 89.6 ± 17.8%. All were PI and receiving pancreatic replacement therapy and nebulised DNase. All were receiving multivitamin supplements, nutritional drinks, inhaled sodium chloride. Those with *P. aeruginosa* were receiving 250 mg azithromycin 3 days per week.	11 HCP (M = 45.5%; 11.3 ± 4.5 y; BMI 16.7 ± 1.5 kg/m^2^) who were recruited as outpatients without chronic disease. FEV_1_ was 96.5 ± 12.1% predicted and FEV_1_/FVC ratio was 107.5 ± 5.9%.	Plasma advances glycation end-products (AGE), amadori products, advanced oxidation protein products (AOPP), dityrosine, formylkynurenine, kynurenine, protein carbonyls, thiol groups and tryptophan. Erythrocyte CAT, glutathione S-transferase (GST), SOD and TAC.
Ambroszkiewicz [60], cross-sectional	Warsaw, Poland	35 people with CF (M = 48.6%; median (range), 7.0 (5–9) y); 60.0% were F508del homozygous; 29.0% were F508del heterozygous; 11.0% were non-F508del. Mean ± SD FEV_1_ was 89.6 ± 12.2% predicted. No participants had CFRD and 2.9% had hepatic insufficiency; 94.3% of participants were PI, and were receiving pancreatic enzyme replacement supplements (6000 U lipase/kg/day). No participants received inhaled or systemic corticosteroids 1 month prior to sample collection. All participants were supplemented with Vitamins A (2000 IU/day), E (200 IU/day) and D3 (400 IU/day)	35 HCP (M = 48.6%; median (range), 7.0 (5–9) y) who were matched for age and sex. HCP were recruited from an outpatient clinic and presented with minor problems other than infections and diseases which may influence bone status.	Serum 25(OH)D, and vitamins A and E.
Olveira [52], cross-sectional	Malaga, Spain	36 people with CF (M = 50.0%; 27.2 ± 8.9 y). Mean ± SD FEV_1_ was 57.8 ± 25.4% and FVC was 68.2 ± 22.8%; 86.1 were colonized with *P. aeruginosa* and mean ± SD exacerbations for the past year was 2.4 ± 2.0. None were receiving Omega-3 fatty acid supplements.	50 HCP (M = 25.0%; 12.8 ± 7.6 y); 54 non-CF bronchiectasis (M = 29.7%; 47.4 ± 18.9 y).	Plasma or serum copper, selenium and vitamin C. Plasma 8-iso-PG2α, CAT, GPx, SOD, TAC and TBARS. Serum vitamins A, D, E:cholerterol and zinc. Neutrophil, lymphocyte, monocyte and total leukocyte peroxide, superoxide and reduced glutathione (GSH).
Bernardi [39], cross-sectional	Sao Paulo, Brazil	44 people with CF (M = 50.0%; 8.4 ± 3.2 y); 31.8% were F508del homozygous; 68.2% for F508del heterozygous; 100% were PI; 6.8% had CFRD; 38.6% had hepatic disease. Furthermore, 25.0% were colonized with *S. aureus* and 6.8% were colonized with *P. aeruginosa*.	16 HCP (M = 25.0%; 8.3 ± 2.6 y).	Erythrocyte GSH.
Sadowska-Woda [72], AquADEK supplementation non-randomized controlled trial	Rzeszow, Poland	50 people with CF (M = 60.0%; 9.6 ± 3.7 y; BMI 19.3 ± 2.8 kg/m^2^). Mean ± SD FEV_1_ was 57.8 ± 25.4% predicted and FVC was 68.2 ± 22.8%; 100.0% were PI and receiving pancreatic enzyme replacement supplements (Creon or Lapancrea). Children were receiving daily inhaled DNase.	21 HCP (M = 66.7%; 9.6 ± 3.1 y; BMI 20.1 ± 5.2 kg/m^2^) who were not receiving multivitamin supplementation. Mean ± SD FEV_1_ 104.2 ± 12.3% predicted and FEV_1_/FVC was 90.0 ± 12.7%.	Plasma hydroperoxides, MDA, thiol groups and TAC. Erythrocyte CAT, SOD and thiol groups.
Cobanoglu [66], cross-sectional	Ankara, Turkey	16 people with CF (M = 50.0%; 6.1 ± 1.5 y and pre-pubertal); 6.3% were F508del homozygous; 43.8% were F508del heterozygous; 50.0% were non-F508del; 100.0% were PI and receiving pancreatic enzyme replacement supplements and 800 IU/day of vitamin D. Non-had CFRD or liver disease. None were receiving anti-epileptic drugs, calcium supplements, systemic or inhaled steroids.	16 HCP (M = 50.0%; 6.1 ± 1.4 y) admitted to hospital for minor issues other than infection and were pre-pubertal.	Serum 25(OH)D.
Durieu [43], intravenous fish oil n-3 emulsion open-pilot observation	Lyon, France	13 people with CF (M = NR; 19.4 ± 11.1 y) prior to treatment with intravenous n-3 fatty acids. Mean ± SD FEV_1_ was 81.5 ± 12.8% and 51.0 ± 15.6% for children and adults, respectively.	21 HCP (M = NR; age range, 20–55 y) from a related study [141].	Plasma hydroperoxides. Plasma vitamins A and E, carotenoids and MDA in CF group only.
van Biervliet [142], cross-sectional	Ghent, Belgium	104 people with CF; separated into two groups, (A) class I, II, III (M = 50.6%; median (IQR), 15.0 (13.4) y), and (B) class IV, V or unknown CFTR genotypes (M = 56.0%; median (IQR), 16.0 (20.0) y). Group A: median (IQR) FEV_1_ was 80.4 (40.4)% predicted and FVC 89.7 (21.3)% predicted; 100% were PI; 11.4% had CFRD; 16.5% had liver disease. Group B: median (IQR) FEV1 was 75.0 (41.4)% and FVC 86.7 (21.3)% predicted; 32.0% were PI; 19.0% had CFRD; 8.0% had liver disease. None received polyunsaturated fat supplements. PI participants received pancreatic enzyme replacement and multivitamin supplements (1000 IU cholecalciferol, 100 mg α-tocopherolacetate, 1 mg phytoimenadion and 10000 IU retionolacetate).	44 HCP (M = NR; median (range), 18.0 (1–47) y).	Serum α-linolenic acid, arachidonic acid, docosahexaenoic acid, linoleic acid and oleic acid.
Rovner [67], cross-sectional	Pennsylvania, USA	101 people with CF (M = 50.0%; 14.8 ± 4.2 y). FEV_1_ was 84 ± 19% predicted. None had CFRD. All participants were receiving vitamin D and pancreatic enzyme supplements.	177 HCP (M = 42.0; 12.5 ± 3.5 y).	Serum 1,25(OH)_2_D and 25(OH)D.
Oudshoorn [63], cross-sectional	Utrecht, The Netherland	30 people with CF (M = 53.3; 11.9 ± 2.6 y, range 8–18 y). FEV_1_ was 88.5 ± 18.7% predicted. All received a mean ± SD 120 ± 75 mg/day α-tocopherol supplementation.	30 outpatients (M = 43.3; 11.3 ± 2.9 y) who underwent ear, nose or throat surgery.	Plasma α-tocopherol, coenzyme Q10:cholesterol ratio, total, oxidized, reduced coenzyme Q10.
Tirouvanziam [40], non-randomized trial of N-acetylcysteine	Stanford, USA	18 people with CF (sex and age NR) prior to treatment with N-acetylcysteine.	9 HCP (sex and age NR).	Neutrophil GSH.
Nicolaidou [44], cross-sectional	Athens, Greece	25 people with CF (M = 50.0; range 6–17 y, median (p25, p75), M = 15.0 (14.0, 16.0), F = 14.5 (11.0, 15.0) y) without a vitamin K intervention. Median (p25, p75) FEV1 was 71.5 (45, 85)% predicted for males and 75.5 (62, 104)% predicted for females. No group specific genotype data was available. 100.0% were PI and receiving pancreatic enzyme replacement supplements. None were receiving antibiotics or had ever received corticosteroids. All were receiving 800 IU/day vitamin D	25 HCP (M = 52.0; range 8–17 y, median (p25, p75), M = 14.0 (12.0, 16.0), F = 11.0 (9.0, 14.0) y).	Serum 25(OH)D and vitamin K.
Back [42], cross-sectional	Tubingen, Germany	22 people with CF (M = 40.9%); separated into 3 groups for analysis: those who are 7 6–11 y (median (IQR), 9.4 (7.7–11.1) y), 7 12–17 y (median (IQR), 15.0 (14.0–16.7) y) or 7 ≥ 18 y (median (IQR), 23.6 (19.7–29.0) y); 27.3% were F508del homozygous; 45.5% were F508del heterozygous; 13.6% were non-F508del. 86.4% were PI and 18.2% had CFRD. Median (IQR) FEV_1_ was 90.0 (84.5–102.0)% predicted in those 6–11 y, 80.0 (63.0–82.0)% predicted in those 12–17 y and 65.0 (42.0–84.0)% predicted in those ≥18 y. FVC was 92.0 (87.0–95.0)% predicted in those 6–11 y, 81.0 (71.0–86.0)% predicted in those 12–17 y and 6588 (65.0–95.0)% predicted in those ≥18 y.	30 HCP (M = 53.3); separated into 3 groups for analysis: 9 6–11 y (median (IQR), 8.4 (7.4–10.3) y), 5 12–17 y (median (IQR), 13.3 (12.6–16.4) y) and 16 ≥ 18 y (median (IQR), 27.6 (24.5–30.2) y).	Plasma vitamin E, β-carotene, β-cryptoxanthin, lycopene, protein carbonyls, TBARS and vitamin C.
Best [71], randomized controlled trial of copper supplementation	Columbus, USA	38 people with CF (M = NR; 24.8 ± 8.0 y, range 12–48 y) before supplementation with copper and zinc; 100% had PI and were receiving pancreatic enzyme replacement and multivitamin supplements (ADEK). None had CFRD, renal failure or advanced lung disease.	30 HCP (age and sex NR).	Erythrocyte SOD. Plasma ceruloplasmin and diamine oxidase
Lagrange-Puget [7], cross-sectional	Lyon, France	Up to 232 people (depending on biomarker studied) with CF (M = NR; mean (range), 13.0 (0.5–45.0) y). All had PI and were receiving pancreatic enzyme replacement supplements. Those with vitamin A and E deficiency received retinol or tocopherol supplements.	53 HCP (M = NR; mean (range), 22.0 (1.0–40.0) y); including, 21 children admitted for orthopedic surgery (before treatment) and 32 healthy individuals attending a phase I clinical trial centre.	Plasma α-carotene, β-carotene, GSH, *t*GSH, lipid peroxides, lutein, lycopene, MDA, TBARS, vitamins A and E, and zeaxanthin.
Schupp [57], cross-sectional	California, USA	10 people with CF (M = 90.0; 31.2 ± 10.0 y, range 21–47 y). Mean ± SD FEV_1_ was 45.0 ± 25.4% predicted. 100% were PI and receiving pancreatic enzyme replacement supplements. 30% had CFRD. All were receiving nutritional supplements, however, 10% were receiving multivitamin supplements containing lutein.	10 HCP (age range 20–51 y) matched for age, sex and ethnicity.	Plasma lutein and zeaxanthin.
Sidlova [88], cross-sectional	Prague, Czech Republic	37 people with CF (M = 64.9; mean (range), 10.4 (1–28) y); 10.8% had hepatobiliary abnormalities.	27 controls (M = 66.7; mean (range), 8.5 (2–17) y).	Serum GSTα
Aris [68], cross-sectional	North Carolina, USA	50 people with CF (M = 46.0; 28.3 ± 7.8 y); 56% were F508del homozygous; 28% were F508del heterozygous; 16% were non-F508del. Mean ± SD FEV1 was 46.1 ± 18.6% predicted and FVC was 67.5 ± 17.9% predicted. 94% were PI and were receiving pancreatic enzyme replacement supplements; All were receiving multivitamin supplementation.	53 HCP (M = 50.9; 28.9 ± 7.8 y) who were matched for age and sex.	Serum 1,25(OH)_2_D and 25(OH)D.
McGrath [54], cross-sectional	Belfast, UK	11 people with CF (M = 72.7%; range, 18–37 y). All were receiving vitamin E supplementation (200 mg/day). None receiving any other antioxidants and all non-smokers.	11 HCP (M = 72.7%; range, 20–35 y). All non-smokers.	Plasma vitamin E:cholesterol, MDA:cholesterol, protein carbonyls and protein thiols.
Wood [41], cross-sectional	Newcastle, Australia	21 people with CF (M = 61.9; 14.8 ± 1.1 y); 57% were F508del homozygous; 38% were F508del heterozygous; 5% were non F508del. Mean ± SEM FEV_1_ was 85.3 ± 6.2% predicted and FVC was 90.1 ± 4.7% predicted. 91% were PI; 71% were receiving aerosol β_2_-agonists; 33% were receiving inhaled corticosteroids; 14% were receiving cromoglycate; 24% were receiving ipratropium; 19% were receiving DNase; 19% were receiving antibiotics. None received vitamin supplementation in the previous 4 wk.	21 HCP (M = 61.9; 14.2 ± 1.1 y) matched for age and sex.	Erythrocyte SOD. Plasma 8-iso-PGF2α, β-carotene, GPx, selenium, zinc, copper, vitamins A, C and E.
Madarasi [61], cross-sectional	Budapest, Hungary	21 people with CF (M = 57.1; mean (range), 8.7 (6–12) y). None had liver manifestations; 100.0% received pancreatic enzyme replacement and multivitamin supplements.	24 HCP (M = 45.8; mean (range), 8.3 (6–12) y) matched for age. HCP were formally hospitalised children with minor ailments who were attending clinic for follow-up blood testing.	Erythrocyte CAT and SOD. Serum α-tocopherol, ascorbic acid, MDA, TAC, uric acid and vitamin A. Whole blood GPx.
Lands [73], cross-sectional	Montreal, Canada	24 people with CF (M = 62.5; 11.4 ± 3.4 y), who were not hospitalised for an acute respiratory exacerbation. Mean ± SD FEV_1_ was 77.6 ± 17.4% predicted and RV/TLV was 31.1 ± 10.3%.	17 HCP (M = 58.8; 23.8 ± 3.9 y) who were recruited as part of a study investigating oxidative stress and exercise.	Plasma trolox equivalent antioxidant capacity (TEAC).
Percival [70], cross-sectional	Florida, USA	7 people with CF (M = 100.0; 24.5 ± 3.5 y, range 19–32 y). Mean ± SD FEV_1_ was 55.0 ± 28.8% (range 25–99%) and FVC was 75.8 ± 20.2% (51–105%) predicted. 85.7% were PI and receiving pancreatic enzyme replacement supplements. All were receiving multivitamin supplements.	6 HCP (M = 100.0; 24.5 ± 5.5 y, range 20–30 y) who were matched for age.	Plasma, polymorphonuclear cell and mononuclear cell copper. Polymorphonuclear cell and mononuclear cell Copper–Zinc SOD. Plasma ceruloplasmin.
Tauber [143], cross-sectional	Vienna, Austria	28 people with CF (M = 51.1; 10.8 ± 6.5 y; this includes those with an acute respiratory exacerbation (*n* = 17)) who did not have an acute respiratory exacerbation; 4.4% of all (including those with an acute respiratory exacerbation) had chronic *P. aeruginosa* infections.	175 children with bronchial asthma (BA) (M = 55.4; 9.8 ± 3.8 y); 87 HCP (M = 29.9; 10.2 ± 4.5 y); 23 non-asthmatic children with bacterial lower respiratory tract infection (LRTI) (M = 39.1; 8.6 ± 2.5 y).	Serum myeloperoxidase (MPO).
Collins [75], cross-sectional	New South Wales, Australia	10 people with CF (sex NR; mean (range), 17.4 (5.8–37.8) y; this includes those with an acute respiratory exacerbation (*n* = 12)), who are not undergoing treatment for an acute respiratory exacerbation.	9 HCP (sex NR; mean (range), 29.8 (22.8–46.1) y).	Plasma 8-iso-PGF2α.
Eichler [144], cross-sectional	Vienna, Austria	23 people with CF (M = 57.1; median (range), 14.5 (8–17) y; this includes those with an acute respiratory exacerbation (*n* = 19)). In those who were clinically-stable, median (IQR) FEV_1_ was 81.2% (72.4–98.4) and FVC was 87.8% (79.6–102.3%) predicted.	25 HCP (M = 56.0; median (range), 13.4 (3–16) y).	Serum lactoferrin and MPO.
Dominguez [64], cross-sectional	Barcelona, Spain	101 people with CF (M = 54.5%; 11.5 ± 6.9 y).	43-95 HCP (varies between biomarkers) of a similar age to the CF group (age and sex NR).	Erythrocyte SOD. Plasma α-tocopherol, glutathione reductase (GR), hydroperoxides, MDA and protein carbonyls.
Koller [145], cross-sectional	Vienna, Austria	23 people with CF (M = 57.1; median (range), 14.5 (8–17) y; this includes those with an acute respiratory exacerbation (*n* = 19)), without an acute respiratory exacerbation. Median (quartile 1–3) FEV_1_ was 81.2 (72.4–98.4)% and FVC was 87.8 (79.6–102.3)%.	25 HCP (M = 56.0; median (range), 13.4 (3–16) y).	Serum eosinophil peroxidase.
Hung [89], cross-sectional	Edinburgh, UK	63 people with CF (M = 49.2; mean (range) 7.9 (0.5–16) y). All participants were receiving pancreatic enzyme replacement and multivitamin supplements; all participants were receiving flucloxacillin, inhaled steroids and/or inhaled steroid and bronchodilators.	59 HCP (M = 62.7; mean (range), 7.2 (1–16) y). 96.6% were screened for CF and fragile X syndrome but had normal genotypes; 1.7% had bronchiolitis; 1.7% had an inguinal hernia.	Serum GSTα_1_.
Kearns [46], cross-sectional	Arkansas, USA	15 people with CF (M = 46.7%; 13.1 ± 2.7 y, range 8.7–19.3 y); 7/15 had genotype analysis; 100% were F508del homozygous; 13.3% were receiving pancreatic enzyme replacement and multivitamin supplements.	15 HCP (M = 46.7%; 14.5 ± 3.1 y, range 8.9–18.9 y).	Serum albumin and (γ-glutamyl transpeptidase) GGT.
Koller [146], cross-sectional	Vienna, Austria	59 people with CF (M = 45.9%; 11.0 ± 7.7 y; this includes those with an acute respiratory exacerbation (*n* = 39)) without an acute respiratory exacerbation.	85 HCP (M = NR; 10.8 ± 5.7 y).	Serum MPO.
Winklehofer-Roob [56], pre-β-alanine intervention	Zurich, Switzerland	Up to 32 people with CF (M = 48.6%; 10.8 ± 7.6 y); 100.0 were PI and receiving pancreatic enzyme replacement and multivitamin supplements.	Up to 40 HCP (M = 35.7%; 31.5 ± 8.0 y).	LDL α-tocopherol and β-carotene. Plasma α-tocopherol, β-carotene and MDA.
Mocchegiani [47], cross-sectional	Ancona, Italy	15 people with CF (M = 46.7%; mean (range), 6.7 (2–13) y); 90% were colonized with *P. aeruginosa*. All were receiving pancreatic enzyme replacement and vitamin supplementation. No individuals with signs of diabetes or heart failure were included. All were receiving antibiotics and bronchodilators.	15 HCP (M = 60.0%; mean (range), NR (2-13) y) who were admitted to hospital for minor surgery.	Serum albumin. Plasma Zinc.
Koller [147], cross-sectional	Vienna, Austria	42 people with CF (M = 47.6%; mean (range), 14.5 (0.8–28) y). Mean ± SD FEV_1_ was 67.8 ± 32.4% predicted (range 17.1–125.5% predicted), MEF_50_ was 52.0 (42.5)% predicted (range 4.4–130.2% predicted). 73.3% were colonized with *P. aeruginosa*.	30 HCP (M = 53.3%; mean (range), 13.4 (4–32) y).	Serum MPO.
Mangione [51], cross-sectional	Pennsylvania, USA	32 people with CF (age and sex NR).	8 HCP who were healthy, active, non-smokers and age-matched to their CF-counterparts.	Erythrocyte GSH.
Vaisman [62], cross-sectional	Rehovot, Israel	11 people with CF (mean (range), NR (4-14) y). “Most” were colonized with *P. aeruginosa*. All were receiving vitamin A and E supplements (100 IU) and multivitamin preparations.	10 age-matched HCP (age and sex NR). Not receiving any vitamin supplementation.	Plasma tocopherol vitamin A. Blood neutrophil tocopherol.
James [38], cross-sectional	Cardiff, UK	22 people with CF (M = 45.5%; median (range), 8.3 (1.6–16.5) y).	9 HCP (M = 22.2%; median (range), 10.9 (4.7–16.4) y).	Plasma and erythrocyte α-tocopherol. Serum selenium.
Stead [50], cross-sectional	London, UK	31 people with CF (24.5 y (range, 17–52 y)); 93.5% receiving pancreatic enzyme replacement and no evidence of liver disease. FEV_1_ was 41.5 ± 11.4% predicted. 96.8% taking vitamin D supplements (daily intake range: 0.6–54.3 *μ*g [24–2172 IU)].	28 HCP (M = NR; age = NR).	Serum 25(OH)D, and 1,25(OH)_2_D. Plasma albumin.
Reiter [65], cross-sectional	Massachusetts and Arizona, USA	20 people with CF (M = 50.0%; range, 12–25 y); 50% in Massachusetts and 50% in Arizona. All were receiving pancreatic enzyme replacement, vitamin supplementation and intermittent antibiotic therapy.	8 HCP (M = 40.0%; 18.6 ± 8.1 y).	Serum carotene, albumin and 1,25(OH)_2_D.
Hahn [48], cross-sectional	St. Louis, USA	21 people with CF (M = 57.1%; mean (range), 20.9 (12–36) y). All were PI. All were receiving pancreatic enzyme replacement (given as Cotazyme) and multivitamin supplements (containing 400 units of vitamin D/tablet). One patient had diabetes and was receiving oral hypoglycaemic agents.	21 age- and sex-matched HCP (M = 57.1%; mean (range), 20.3 (12–34) y).	Serum 25(OH)D, albumin and carotene.
Jacob [49], cross-sectional	Chicago, USA	18 people with CF (range, 6–17 y). All were receiving daily multivitamin supplements (Poly-vi-sol) containing 2500 IU of water miscible vitamin A per tablet.	40 adolescent HCP (range, 11–17 y).	Plasma vitamin A, zinc and albumin.
Hubbard [55], cross-sectional	Tennessee, USA	16 people with CF (M = 43.8%; range, 13–47 y); 68.8% were PI. Those with PI were receiving pancreatic enzyme replacement and multivitamin supplements.	8 HCP (M = 50.0%; range, 19–32 y).	Serum vitamin A and carotene. Plasma vitamin E.

Data is expressed as mean ± standard deviation (SD) unless otherwise stated.

### 2.6 Data collection process

Two review authors (AJC and ZLS) used a review-specific form to independently extract relevant data on study design, study population, medications, eligibility criteria, biomarkers and biological fluids studied, and location. Details of the participants (both case and controls) were recorded where possible, such as information on age, sex, genotype, lung function, co-morbidities and treatments. When available, we extracted data on subgroups, including those stratified by severity, genotype or co-morbidities. Data was extracted from graphs using plot digitizer software. Where data and/or measures of variability were not available within a manuscript, this was either calculated or requested from the corresponding author. If data was not made available, the reference was included in the systematic review but excluded from the meta-analysis.

### 2.7 Assessment of quality and risk of bias of included studies

Two review authors (AJC and ZLS) independently assessed the quality of studies using the Newcastle-Ottawa Quality Assessment Scale (NOS) for Case-Control Studies [34]. To minimize bias in the interpretation of the scale, both authors independently assessed 10 unrelated studies not included in the present review, whereby disparities in judgements were discussed and a consensus was reached prior to the assessment of studies included in the present review. Each study was graded for selection, comparability and exposure, and could be awarded a maximum of 9 points. Studies scoring 0–2 were considered low quality, studies scoring 3–5 were considered moderate quality and studies scoring 6–9 points were considered high quality [33]. Risk of publication bias was assessed at the outcome level using a funnel plot [35], which was tested for asymmetry. These methodologies have been used in similar meta-analyses [31,33].

### 2.8 Measures of the group effect

Measures of the group effect were calculated using the RevMan v5.3 (Cochrane, UK) software [36]. For each study, mean differences and 95% confidence intervals (CI) were calculated [36]. Given that biomarkers were consistently expressed to different units or analysed using different methodologies, standardized mean differences (SMD) were calculated.

### 2.9 Assessment of heterogeneity in included studies

Assessments of heterogeneity were calculated using the RevMan v5.3 (Cochrane, UK) software [36]. In the present review, the *I*^2^ statistic was used to assess heterogeneity. The *I*^2^ statistic provides a percentage value that represents the variability in effect estimates that is due to heterogeneity. An *I*^2^ of ≥75% suggests considerable heterogeneity [36]. In the presence of considerable heterogeneity, which could be explained by methodological or population-based considerations, a random-effects model was incorporated using The DerSimonian-Laird method [37]. Data of considerable heterogeneity that could not be explained by methodological or population-based considerations were excluded from the meta-analysis. If heterogeneity could not be explained by methodological or clinical factors, data were not pooled but, instead, reported within the text. These techniques have been used in similar meta-analyses [31,33].

### 2.10 Data synthesis

Results of comparable studies were pooled using either fixed-effect or random-effects models, dependent upon the extent of heterogeneity.

## 3 Results

### 3.1 Study selection

**Fig. 1:**
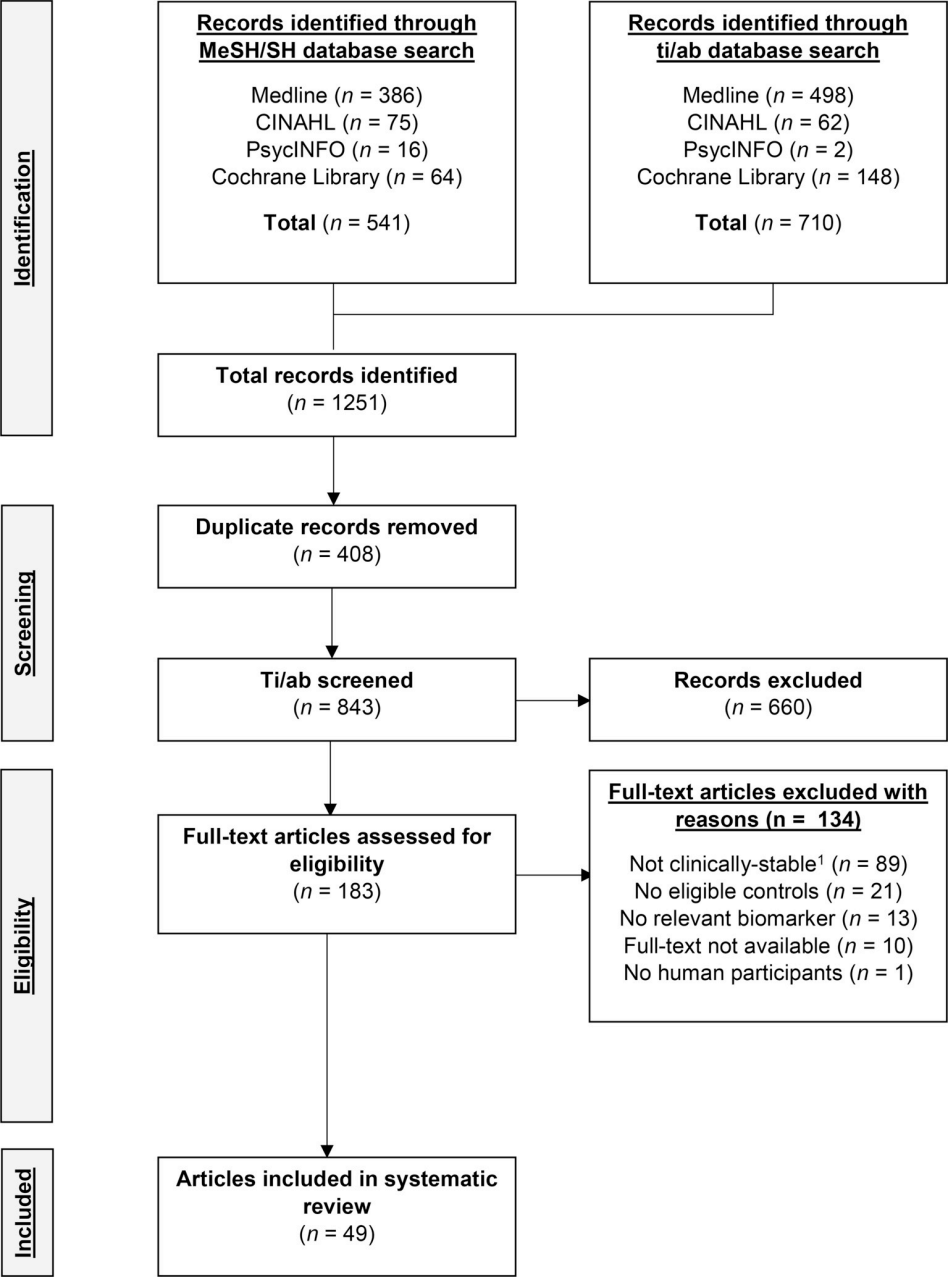
A CONSORT flow diagram of the study identification, screening and selection process. ^1^ Excluded studies either investigated participants who are not clinically-stable (*n* = 12) or does not mention the status of participants (*n* = 77). MeSH, Medical Subject Headings; SH, Subject heading; Ti/ab, title and abstracts.

A flow diagram of the screening process is presented in Fig. 1. Briefly, after the removal of duplicate references, a total of 843 references were screened for their eligibility for the present systematic review. Of the 843 references, 660 were excluded during the ti/ab screen as they were outside of the scope of the present review. Following the screening of the full-texts (*n* = 183; *n* = 10 were excluded as the full-text was not available in English or French), a further 124 references were excluded because cases were not classified as clinically-stable (*n* = 89), there was no eligible control group (*n* = 21), the study did not include biomarkers which were within the scope of the present review (*n* = 13) or the study was not conducted in humans (*n* = 1). Consequently, 49 references were eligible for inclusion in the present review including a total of 1792 people with CF and 1675 controls, in which 25 biomarkers were eligible for meta-analysis (Table 1).

### 3.2 Study characteristics

A summary of the included references are presented in Table 1. The included studies were published between 1977 and 2017, and data were collected from 28 centres in Europe, 15 centres in North America, 3 centres in Asia, 2 centres in Oceania and 1 centre in South America. The majority of studies were of cross-sectional study design (*n* = 43; 87.8%), along with data collected at baseline in 2 non-randomized controlled trials (4.1%), 2 non-randomized and non-controlled trials (4.1%), 1 randomized controlled trial (2.0%), and 1 open-pilot observation (2.0%; Table 1). The sample sizes of included studies were generally modest, with the number of cases and controls ranging from 7 to 232 and 6 to 177, respectively.

### 3.3 Risk of bias and quality of studies

A summary of the risk of bias assessments is presented in Table 2. Briefly, the quality of the studies was generally moderate (*n* = 31; 63.3%), with 14 high (28.6%) and 4 low (8.2%) quality studies included.

**Table 2:** Summary of the quality ratings for the eligible studies using the Newcastle-Ottawa Quality Assessment Scale for case-control studies.

First author [reference]	Selection	Comparability	Exposure	Total points	Rating^a^
AbdulWahab [69]	2	1	3	6	High
Ambroszkiewicz [60]	3	2	2	7	High
Antus [74]	3	0	1	4	Moderate
Aris [68]	2	1	3	6	High
Back [42]	1	1	2	4	Moderate
Bernardi [39]	2	1	2	5	Moderate
Best [71]	1	1	3	5	Moderate
Cobanoglu [66]	2	2	3	7	High
Collins [75]	3	0	3	6	High
Dominguez [64]	1	1	1	3	Moderate
Durieu [43]	0	0	2	2	Low
Eichler [144]	2	0	1	3	Moderate
Hahn [48]	2	2	3	7	High
Hubbard [55]	2	0	1	3	Moderate
Hung [89]	3	0	1	4	Moderate
Jacob [49]	2	0	2	4	Moderate
James [38]	0	0	2	2	Low
Kearns [46]	1	1	2	4	Moderate
Koller [145]	1	0	2	3	Moderate
Koller [146]	0	0	2	2	Low
Koller [147]	1	0	2	3	Moderate
Konstantinidis [139]	1	1	3	5	Moderate
Lagrange-Puget [7]	2	0	2	4	Moderate
Lands [73]	1	0	2	3	Moderate
Lee [45]	2	0	1	3	Moderate
Madarasi [61]	1	1	2	4	Moderate
Mangione [51]	0	1	3	4	Moderate
McGrath [54]	3	2	3	8	High
Mocchegiani [47]	1	1	3	5	Moderate
Nicolaidou [44]	2	0	3	5	Moderate
Olveira [58]	1	2	3	6	High
Olveira [52]	0	1	3	4	Moderate
Oudshoorn [63]	0	1	3	4	Moderate
Percival [70]	3	1	3	7	High
Reiter [65]	3	2	3	8	High
Rovner [67]	3	0	3	6	High
Sadowska-Bartosz [53]	1	0	3	4	Moderate
Sadowska-Woda [72]	0	1	3	4	Moderate
Schupp [57]	2	2	3	7	High
Sidlova [88]	2	0	3	5	Moderate
Stead [50]	2	1	2	5	Moderate
Tauber [143]	0	1	2	3	Moderate
Tirouvanziam [40]	1	0	3	4	Moderate
Turowski [140]	2	0	3	5	Moderate
Vaisman [62]	0	1	3	4	Moderate
Van Biervliet [142]	2	0	3	5	Moderate
Winklhofer-Roob [56]	2	0	2	4	Moderate
Wood [41]	4	2	3	9	High
Yadav [59]	2	1	3	6	High

a Studies scoring 0–2 were considered low quality, studies scoring 3–5 were considered moderate quality and studies scoring 6–8 points were considered high quality.

### 3.4 Missing data

Missing data was requested for 7 studies as the mean and/or standard deviation (SD) were not computable [[38], [39], [40], [41], [42], [43], [44]]; however, no authors provided additional data.

### 3.5 Synthesis of results

#### 3.5.1 Thiols

Twelve eligible studies assayed the content of thiols within the erythrocytes, blood neutrophils, plasma or serum of people in clinically-stable CF versus non-CF controls. The biomarkers studied were plasma or serum albumin (*n* = 6) [[45], [46], [47], [48], [49], [50]], erythrocyte GSH (*n* = 2) [39,51], blood neutrophil GSH (*n* = 2) [40,52] and plasma or serum protein thiol groups (n = 2) [53,54].

Whilst GSH was studied in blood neutrophils (*n* = 2) [40,52] and erythrocytes (*n* = 2) [39,51] by 4 eligible trials, a meta-analysis was not possible for either biomarker as mean ± SD was not computable for 2 trials as medians and interquartile ranges were reported [39,40]. The concentration of albumin in the plasma or serum was significantly lower in people with clinically-stable CF compared to the non-CF controls (SMD -0.98, 95% CI -1.68 to −0.27, *p* < 0.01, *I*^2^ 86%, 6 trials) [[45], [46], [47], [48], [49], [50]]. The concentration of protein thiol groups in plasma or serum were not significantly different between people with clinically-stable CF and non-CF controls (SMD -0.35, 95% CI -0.90 to 0.20, *p* = *0.22*, *I*^2^ 0%, 2 trials) [53,54].

#### 3.5.2 Vitamins

Twenty-two studies were eligible and studied the levels of vitamins within the plasma or serum of people with clinically-stable CF versus non-CF controls. The most studied biomarkers were related to the fat-soluble vitamins A (inclusive of ‘vitamin A’, ‘retinol’, carotene, β-carotene, lutein and lycopene; *n* = 13) [7,41,42,48,49,[55], [56], [57], [58], [59], [60], [61], [62]], E (inclusive of ‘vitamin E’, ‘vitamin E:cholesterol’, tocopherol and α-tocopherol; *n* = 13) [7,38,41,42,52,[54], [55], [56],[59], [60], [61], [62], [63], [64]], D (inclusive of ‘vitamin D, ‘25(OH)D’ and ‘1,25(OH)2D’; *n* = 11) [44,45,48,50,[58], [59], [60],[65], [66], [67], [68]] and C (inclusive of ‘vitamin C’, ‘ascorbic acid’ and ‘zeaxanthin’; *n* = 5) [7,41,42,57,61].

**Fig. 2:**
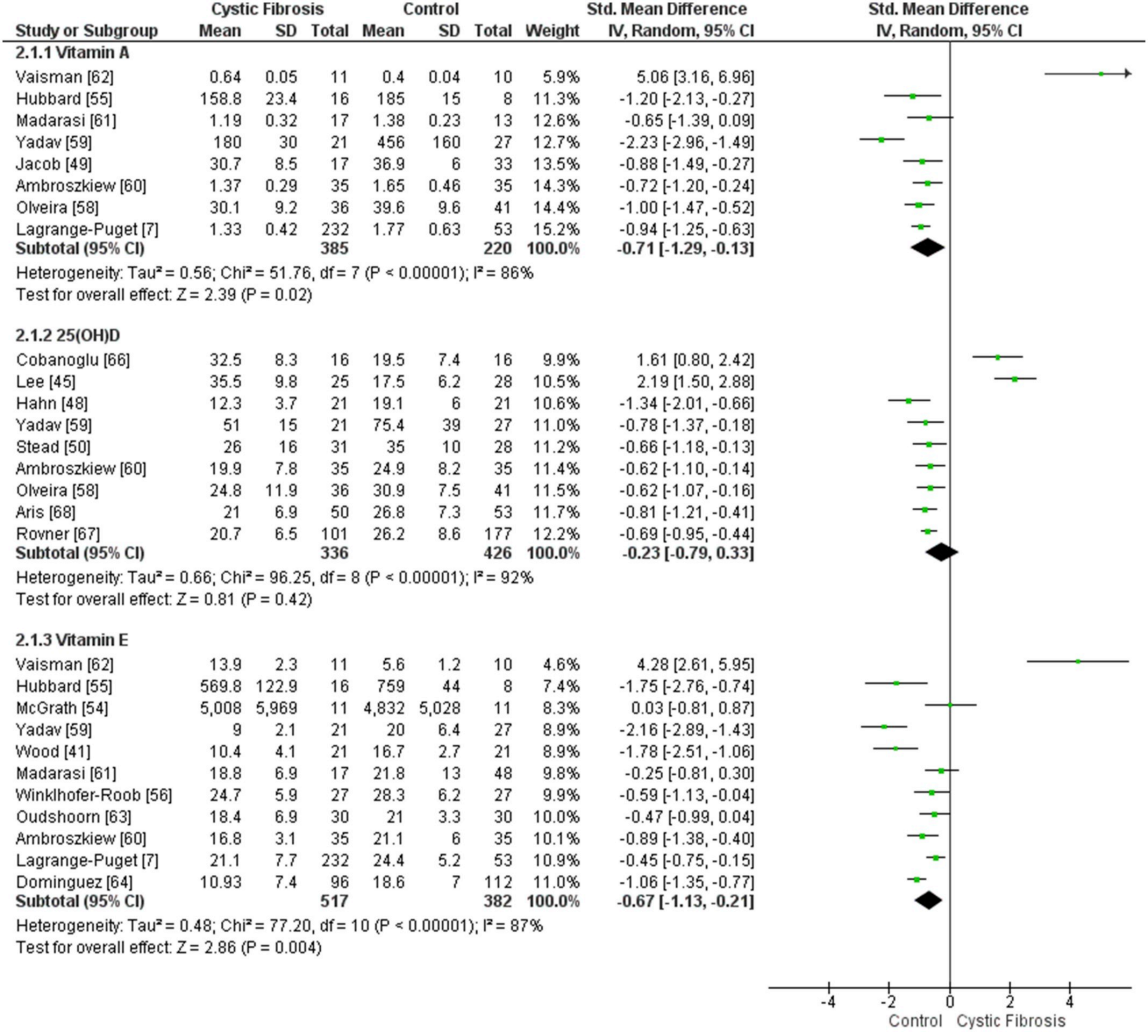
Forest plot demonstrating the differences in fat-soluble vitamin concentrations in clinically-stable people with CF versus non-CF controls. N.b. ‘vitamin A’ is inclusive of ‘retinol’, ‘25(OH)D’ is inclusive of ‘vitamin D’ and ‘vitamin E’ is inclusive of ‘tocopherol’ and ‘α-tocopherol’. 95% CI, 95% confidence interval; IV, inverse variance; SD, standard deviation. N.b. a negative SMD represents a lower vitamin concentration in CF versus controls, whereas a positive SMD represents a higher vitamin concentration in CF versus controls.

The concentration of vitamin A (inclusive of ‘retinol’; SMD -0.66, 95% CI -1.14 to −0.17, *p* = 0.02, *I*^2^ 83%, 8 trials; Fig. 2) [7,41,49,55,[58], [59], [60], [61], [62]] and β-carotene (inclusive of ‘carotene’; SMD -2.17, 95% CI -3.30 to −1.03, *p* = < 0.01, *I*^2^ 91%, 4 trials) [7,48,55,56] were significantly lower in the plasma or serum of people with CF compared to the non-CF controls. The non-provitamin A carotenoids, lutein (SMD -1.52, 95% CI -1.83 to −1.20, *p <* 0.01, *I*^2^ 0%, 2 trials) [7,57], lycopene (2 trials; mean ± SD not computable for 1 trial [42], therefore, meta-analysis was not complete) [7,42] and zeaxanthin (SMD 1.48, 95% CI -5.87 to 8.83, *p* = 0.69, *I*^2^ 99%, 2 trials) [7,57] were also studied in plasma or serum samples. The concentration of vitamin C (inclusive of ‘ascorbic acid’) in the plasma or serum were not significantly different between people with clinically-stable CF and non-CF controls (SMD -0.05, 95% CI -1.83 to 1.72, *p* 0.95, *I*^2^ 93%, 2 trials) [41,61]. The concentration of 25(OH)D (inclusive of ‘vitamin D’) in the plasma or serum were not significantly different between people with clinically-stable CF and non-CF controls (SMD -0.23, 95% CI -0.79 to 0.33, *p* = 0.42, *I*^2^ 92%, 9 trials; Fig. 2) [45,48,50,[58], [59], [60],[66], [67], [68]]. A metabolite of 25(OH)D, 1,25(OH)_2_D, has also been studied, however concentrations in plasma or serum were not different between people with CF and non-CF controls (SMD -0.46, 95% CI -1.12 to 0.20, *p* = 0.17, *I*^2^ 90%, 4 trials) [50,65,67,68]. Data were taken from samples collected during winter months where possible. The concentration of vitamin E (inclusive of ‘tocopherol’ and ‘α-tocopherol’) in the plasma or serum were significantly lowered in people with CF compared to their non-CF controls (SMD -0.67, 95% CI -1.13 to −0.21, *p* < 0.01, *I*^2^ 87%, 11 trials; Fig. 2) [7,41,[54], [55], [56],[59], [60], [61], [62], [63], [64]]. However, plasma or serum vitamin E:cholesterol was not significantly different between people with CF and their non-CF controls (SMD -0.35, 95% CI -0.03 to 0.74, *p* = *0.07, I*^2^ 0%, 2 trials) [52,54].

#### 3.5.3 Trace elements

Eight studies were eligible and studied the levels of trace elements within the plasma or serum of people with CF versus non-CF controls. The trace elements studied were zinc (*n* = 6) [41,47,49,58,59,69], copper (*n* = 3) [41,59,70] and selenium (*n* = 2) [38,41].

The concentration of copper in the plasma or serum were not significantly different between people with clinically-stable CF and non-CF controls (SMD 0.53, 95% CI -0.25 to 1.30, *p* = 0.53, *I*^2^ 67%, 3 trials) [41,59,70]. The concentration of selenium in the plasma or serum were significantly lower in people with CF versus the non-CF controls in 1 trial [41], however, a meta-analysis was not conducted in this biomarker as mean ± SD was not computable for the second trial [38]. The concentration of zinc in the plasma or serum was not significantly different between people with CF and non-CF controls (SMD -0.50, 95% CI -1.21 to 0.20, *p* = 0.16, *I*^2^ 89%, 6 trials) [41,47,49,58,59,69].

#### 3.5.4 Oxidoreductases

Five studies were eligible and studied oxidoreductases within the erythrocytes, plasma or serum of people with CF versus non-CF controls. The oxidoreductases studied were erythrocyte SOD activity (*n* = 5) [41,53,61,64,71], erythrocyte CAT activity (*n* = 2) [53,61] and plasma or serum ceruloplasmin (n = 2) content [70,71].

CAT activity in the erythrocytes were not significantly different between people with CF and non-CF controls (SMD -0.40, 95% CI -1.51 to 0.72, *p* = 0.49, *I*^2^ 78%, 2 trials) [53,61]. Ceruloplasmin content of the plasma or serum were not significantly different between people with CF and non-CF controls (SMD 0.60, 95% CI -0.38 to 1.58, *p* = 0.23, *I*^2^ 58%, 2 trials) [70,71]. SOD activity in the erythrocytes were not significantly different between people with CF and non-CF controls (SMD 0.02, 95% CI -1.16 to 1.20, *p* = 0.97, *I*^2^ 96%, 5 trials) [41,53,61,64,71].

#### 3.5.5 Total antioxidant capacity

Four studies were eligible and studied the total antioxidant capacity (TAC) (inclusive of total antioxidant status [TAS] and trolox equivalent antioxidant status (TEAS)) of plasma or serum in people with CF versus non-CF controls. TAC of the plasma or serum was not significantly different between people with CF compared and the non-CF controls (SMD -0.82, 95% CI -2.02 to 0.37, p = 0.18, *I*^2^ 94%, 4 trials) [58,61,72,73].

#### 3.5.6 Lipid peroxidation

Eleven studies were eligible and studied the levels of lipid peroxidation within the plasma or serum of people with CF versus non-CF controls. The biomarkers studied were MDA (inclusive of the thiobarbitoric acid reactive substances [TBARS] assay) (*n* = 7) [7,42,54,56,58,61,64,72,74], hydroperoxides (*n* = 3) [43,64,72] and total 8-iso-PGF_2α_ (*n* = 3) [41,58,75].

**Fig. 3:**
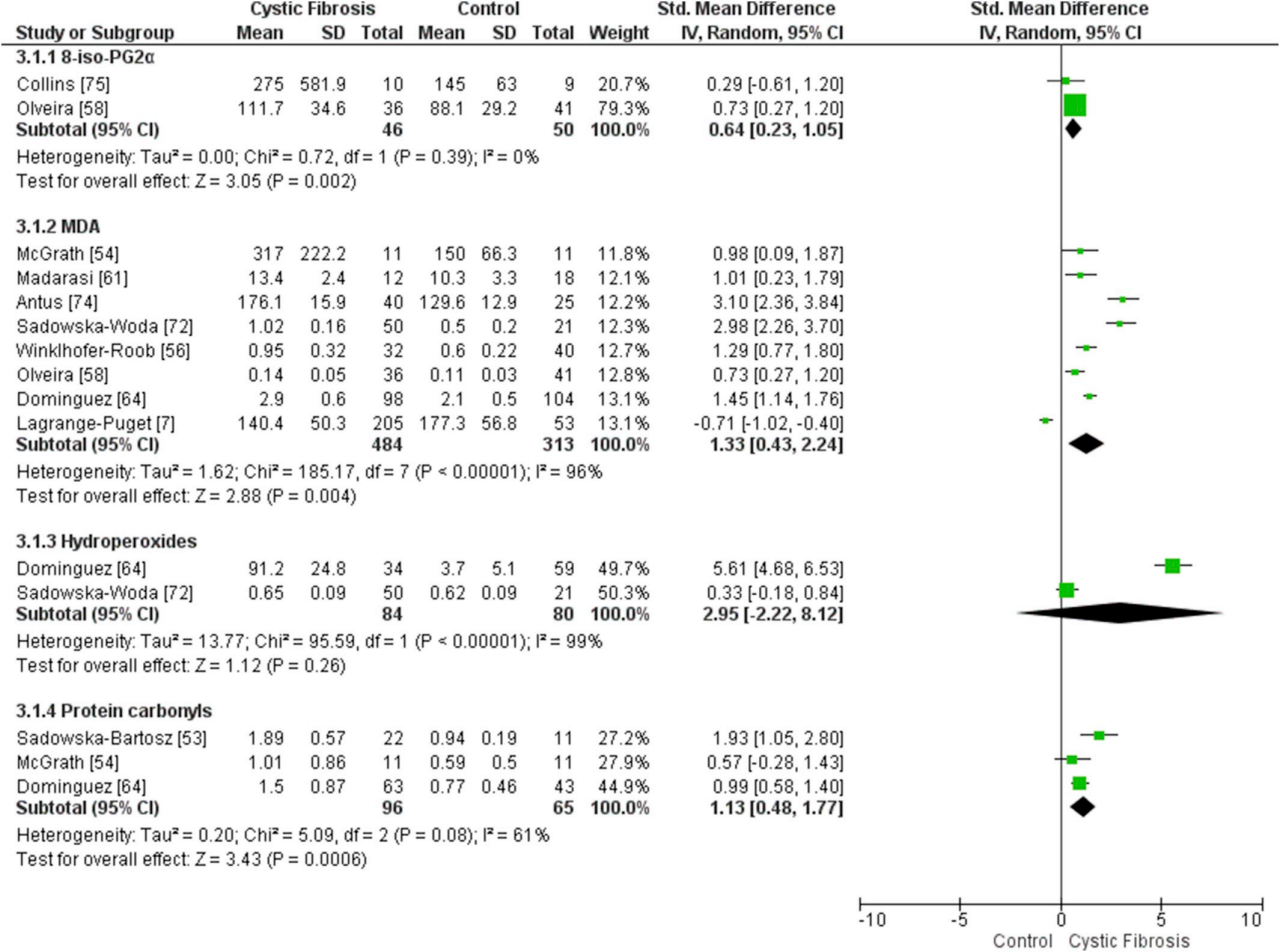
Forest plot demonstrating the differences in biomarkers of oxidative damage in clinically-stable people with CF versus non-CF controls. 95% CI, 95% confidence interval; IV, inverse variance; SD, standard deviation. N.b. a negative SMD represents a lower vitamin concentration in CF versus controls, whereas a positive SMD represents a higher vitamin concentration in CF versus controls.

The concentration of MDA (SMD 1.33, 95% CI 0.43 to 2.24, *p* < 0.01, *I*^2^ 96%, 8 trials; Fig. 3) [7,54,56,58,61,64,72,74] and total 8-iso-PGF_2α_ (SMD 0.64, 95% CI 0.23 to 1.05, *p <* 0.01, *I*^2^ 0%, 2 trials; Fig. 3) [58,75] in the plasma or serum were significantly higher in people with clinically-stable CF compared to the non-CF controls (Fig. 3). The concentration of hydroperoxides (SMD 2.80, 95% CI -2.71 to 8.31, *p* = 0.26, *I*^2^ 99%, 2 trials; Fig. 3) [64,72] in the plasma or serum were not significantly different between people with CF and non-CF controls.

#### 3.5.7 Protein carbonylation

The only biomarker of protein carbonylation studied was plasma or serum protein carbonyls (*n* = 4) [42,53,54,64]. The concentration of protein carbonyls in the plasma or serum were significantly higher in people with CF compared to the non-CF controls (SMD 1.13, 95% CI 0.48 to 1.77, *p* < 0.01, *I*^2^ 61%, 3 trials; Fig. 3) [53,54,64].

## 4 Discussion

This systematic review and meta-analysis is the first to investigate whether circulating biomarkers of antioxidant status and oxidative stress are abnormal in people with clinically-stable CF versus non-CF controls. Forty-nine eligible studies were identified, including a total of 1792 people with CF and 1675 controls, in which 25 biomarkers were eligible for meta-analysis. Two principal findings were observed: (1) vitamins A and E, β-carotene, lutein and albumin were significantly lowered in the plasma or serum of people with clinically-stable CF versus non-CF control participants, and (2) protein carbonyls, total 8-iso-PG2α and MDA were significantly higher in the plasma or serum of people with clinically-stable CF versus non-CF control participants. These data provide systematic evidence that oxidative stress may be implicated in the pathophysiology of CF even in those who are clinically-stable and free from a pulmonary exacerbation at the time of sampling.

### 4.1 Thiols

GSH is a tripeptide compound which plays a major role in regulating intracellular redox balances. In the present review, 4 eligible studies quantified GSH in the erythrocytes [39,51] and blood neutrophils [40,52] of people with CF versus controls, however meta-analyses were not possible due to appropriate data not being reported in 2 of these trials [39,40]. Notably, neither trial included in the present review reported significant differences in erythrocyte GSH in people with CF compared to controls [39,51]; however, a significant negative correlation between erythrocyte GSH and FEV_1_ was reported in adults with mild-to-very severe CF lung disease [51] (Table 3). Mangione et al. [51] speculated that these observations reflected the mobilisation of GSH into circulation to neutralize pulmonary reactive oxygen species in advanced lung disease. Therefore, it could be suggested that elevated erythrocyte GSH is a bookmark for historical incidences of oxidative damage in the CF lung [51]. It is unknown whether other causes of oxidative stress in CF (e.g. CF-related diabetes [CFRD]) contribute to altered erythrocyte GSH concentrations.

**Table 3:** Statistically significant correlation coefficients for biomarkers of antioxidant status, oxidative damage and clinical outcomes.

First author [reference]	Biomarker	Sample		Clinical outcome	r
*Vitamins*					
Lagrange-Puget [7]	Vitamin A	P/S	BMI FEV_1_		+NR +NR
Madarasi [61]	Vitamin A	P/S	Shwachman-Kulzycki score		+0.75
Lagrange-Puget [7]	β-carotene	P/S	BMI FEV_1_		+NR +NR
Wood [41]	β-carotene	P/S	Monocyte count Neutrophil count White cell count		−0.31^a^− 0.46^a^− 0.42^a^
Lagrange-Puget [7]	Lutein	P/S	BMI		+NR
Schupp [57]	Lutein	P/S	Macular pigment optical density		+0.76
Lagrange-Puget [7]	Lycopene	P/S	BMI FEV_1_		+NR +NR
Lagrange-Puget [7]	Zeaxanthin	P/S	FEV_1_		+NR
Schupp [57]	Zeaxanthin	P/S	Macular pigment optical density		+0.80
Madarasi [61]	Vitamin C	P/S	Shwachman-Kulzycki score		+0.49
Back [42]	Vitamin C	P/S	Age FEV_1_		−0.77 +0.50
Aris [68]	Vitamin D	P/S	Serum intact parathyroid hormone Serum osteocalcin Urinary free deoxypyridinoline		-NR +NR -NR
Hahn [48]	Vitamin D	P/S	Serum calcium Diaphyseal bone mass		+0.54 +0.42
Aris [68]	1,25(OH)_2_D	P/S	Serum intact parathyroid hormone Serum osteocalcin Urinary free deoxypyridinoline		-NR +NR -NR
Back [42]	Vitamin E	P/S	Age FEV_1_		−0.47 +0.46
Lagrange-Puget [7]	Vitamin E	P/S	FEV_1_		+NR
Oudshoorn [63]	Vitamin E	P/S	Coenzyme Q_10_		+0.40
Wood [41]	Vitamin E	P/S	Monocyte count Neutrophil count		−0.34^a^− 0.34^a^
*Trace elements*					
AbdulWahab [69]	Zinc	P/S	BMI FEV_1_ FEV_1_/FVC FVC		+NR +NR +NR +NR
Jacob [49]	Zinc	P/S	Age Retinol binding protein Vitamin A		−0.63 +0.64 +0.51
Mocchegiani [47]	Zinc	P/S	Interleukin-2 Weight/age (percentile) Weight/height deficits (%)		+0.79^1^ +0.86^a^ +0.94^a^
*Thiols*					
Mangione [51]	Glutathione	Erythrocyte	FEV_1_ FVC		−0.30 +0.35
*Other antioxidants*					
Lands [73]	TEAC	P/S	BMI FEV_1_ Uric acid Height Weight RV/TLC		+0.47^b^ +0.43^b^ +0.49^b^ +0.39^b^ +0.50^b^− 0.42^b^
*Lipid peroxidation*					
Wood [41]	8-iso-PGF_2α_	P/S	P/S β-carotene P/S vitamin C P/S vitamin E Monocyte count Neutrophil count White cell count		−0.42^a^− 0.43^a^− 0.37^a^ +0.37^a^ +0.31^a^ +0.31^a^
Lagrange-Puget [7]	MDA	P/S	Age		+NR
Lagrange-Puget [7]	TBARS	P/S	Age BMI		+NR -NR

a Denotes that both case and control participants were included in the correlation analysis.

b Denotes that both non-hospitalised and hospitalised participants were included in the correlation analysis. +, statistically significant positive correlation (*p* ≤ 0.05); -, denotes statistically significant negative correlation (*p* ≤ 0.05); 8-iso-PGF_2α_, F_2_-isoprostane 8-iso-prostaglandin F_2α_; BMI, body mass index; FEV_1_, forced expiratory volume in 1 s; MDA, malondialdehyde; NR, correlation coefficient not reported, but there was a statically significant correlation (*p* ≤ 0.05); P/S, plasma or serum; PICP, serum carboxy-terminal propeptide of type I procollagen; PINP, serum amino-terminal propeptide of type I procollagen; *r*, correlation coefficient; RV/TLC, residual volume/total lung capacity; TEAC, trolox equivalent antioxidant capacity; TBARS, Thiobarbituric acid reactive substances.

Functional CFTR is permeable to GSH, and defective or deficient CFTR may contribute to oxidative stress, due to insufficient concentrations of extracellular GSH [12]. No studies were eligible for the present review that quantified plasma GSH, as GSH is rapidly oxidized in plasma to form glutathione disulphide (GSSG) [76]. Two studies demonstrated that there were no differences between plasma protein thiol groups, which are rich in Cys, in people with CF compared to controls [53,54]. Conversely, plasma or serum albumin, a protein which constitutes the majority of the plasma protein thiol pool, was significantly lower in people with CF compared to controls [[45], [46], [47], [48], [49], [50]]. Notably, serum albumin may also be affected in CF by various co-morbidities including liver disease [77] and malnutrition [78]. Importantly, only 2 trials that studied circulating thiols reported the genotype of participants [39] and no trials reported results from a group with a single homozygous genotype. Previous reports from cell culture have shown that GSH efflux is lowered even in genotypes with residual CFTR function (classes IV-VI) [17]; however, no studies to date have investigated whether higher concentrations of extracellular thiols contribute to the improved phenotype often observed in class IV-VI genotypes. It is, therefore, important for future studies to control for the genotype of participants and also investigate differences in thiol concentrations between subgroups classified by genotype.

An important area for future research is to investigate the thiol:disulphide couples in the blood of individuals with clinically-stable CF. For instance, Roum et al. [79] demonstrated that total plasma GSH was ~40% lower in people with CF versus healthy controls, however this study was excluded from the present review as it was unclear whether included cases were clinically-stable. In a later review, Ziady & Hansen [15] suggested that whilst the GSH Eh was not significantly different between people with CF and their healthy counterparts in the study by Roum et al. [79] (−139 mV vs. −138 mV, respectively), the lower concentration of GSH meant that an acute oxidative pertubation would affect GSH Eh to a greater degree in those with CF versus healthy controls [15]. However, to fully understand the redox potential of thiols within plasma of people with CF, other thiol:dilsufide couples must be considered, including Cys [80] and thioredoxin [81]. No studies to date have investigated the Cys:Cyss in people with CF. Importantly, whilst extracellular Cys pools are a key regulator of GSH synthesis, GSH:GSSG and Cys:cystine (Cyss) may have independent functions. For instance, a lowered Cys:Cyss ratio causes mitochondria-derived reactive oxygen species generation, which subsequently activates the key antioxidant transcriptional factor, nuclear factor erythroid 2-related factor 2 (Nrf2) [82]. Interestingly, pharmacological inhibition of CFTR in cellular models causes lowered expression of Nrf2 in the lung epithelium [83]; therefore, thiol perturbations could contribute to the accumulation of cytotoxic reactive oxygen species in CF.

### 4.2 Micronutrients

Important sources of exogenous antioxidants are the consumption of water- and fat-soluble vitamins. In terms of water-soluble micronutrients, vitamin C content was not significantly different between people with CF and controls; however, this was only addressed by 2 trials of moderate-to-high quality [41,61]. Importantly, Madarasi et al. [61] reported significantly elevated vitamin C in children with CF compared to age-matched healthy controls; however, participants enrolled on this study were administered 100–200 mg/d of ascorbic acid. Conversely, Wood et al. [41] reported significantly lowered concentrations of vitamin C in adolescents with CF who refrained from multivitamin supplementation for 4 weeks prior to sampling versus age- and sex-matched healthy controls. Fat-soluble micronutrients, vitamin A [7,41,49,55,[58], [59], [60], [61], [62]], β-carotene [7,48,55,56], lutein [7,57] and vitamin E [7,41,55,56,[59], [60], [61], [62], [63], [64]] were significantly lowered in the plasma or serum of people with CF versus controls, whilst 25(OH)D [45,48,50,[58], [59], [60],[66], [67], [68]], 1,25(OH)_2_D [50,65,67,68] and vitamin E:cholesterol [52,54] were not significantly different. Importantly, there was a considerable degree of heterogeneity in the analysis of both water- and fat-soluble micronutrients, likely caused by differences in the studied population (i.e. age, sex and genotype), differences in measurement units and supplementation protocols (in addition to adherence variability [18]).

Interestingly, Wood et al. [41] found significant negative correlations between lipid peroxidation biomarkers and vitamins C, E, and β-carotene (Table 3). It was speculated that the intensified inflammatory-induced free radical production may result in a depletion of antioxidant defences in non-supplemented CF participants [41]. Furthermore, vitamins C and E were positively correlated with prognostically relevant clinical outcomes, such as FEV_1_ and the Shwachman-Kulzycki score (Table 3). Multivitamin supplementation is a logical strategy to increase antioxidant pools in the blood of people with CF and is recommended by The European Society for Clinical Nutrition and Metabolism [26]. However, increasing the concentrations of antioxidant vitamins is challenging in this population due to suboptimal dosing, poor adherence to treatment or nutrient malabsorption [18]. The high treatment burden of CF, and the subsequent poor adherence to oral supplements, is a considerable challenge when developing interventions to mitigate oxidative stress, which is evidenced by the conflicting evidence regarding micronutrient supplementation for the management of CF lung disease [84].

In addition to water- and fat-soluble vitamins, various trace elements in blood have direct antioxidant abilities as well as mediating the activity of oxidoreductases. Only 8 included studies investigated trace element levels in the blood of people with clinically-stable CF. The concentration of plasma or serum copper [41,59,70] and zinc [41,47,49,58,59,69] were not different between people with CF and controls. Furthermore, whilst it was not possible to conduct meta-analysis on selenium, neither of the included trials reported a significant difference between the CF and control groups [38,41]. Two trials reported correlations between zinc and clinically relevant outcomes, including positive relationships with lung function, nutritional status and interleukin-2 (Table 3) [47,69]. These data suggest that suboptimal zinc content may be observed in those with more severe lung disease and/or malnutrition. However, whether this contributes to cases of oxidative stress is currently unknown and warrants further investigation.

### 4.3 Oxidoreductases

In addition to thiol groups and the exogenous micronutrients, blood also contains antioxidant defence enzymes that have direct antioxidant effects as well as catalysing reactions which maintain thiol:disulfide ratios, which limits the development of oxidative stress [6]. The most studied of these enzymes, SOD, was investigated in 5 trials included in the present systematic review [41,53,61,64,71]. However, meta-analysis demonstrated that there were no significant differences in erythrocyte SOD activity between those with CF and controls [41,53,61,64,71]. Notably, there was a large variability in the results of the included trials, with 2 demonstrating lowered [71,85], 2 indifferent [41,53] and 1 elevated [64] erythrocyte SOD activity in the CF group versus controls. Whilst the cause for such variable results is unknown, the only trial showing elevated erythrocyte SOD activity in CF did not use a methodology that expressed data relative to haemoglobin or red blood cell count [86]. While it was not possible to quantify iron deficiencies in the present review, iron deficiency is reported in 64% of clinically-stable adults with CF [87] and iron supplementation is recommended in those with persistent inflammation [26]. Therefore, it is possible that the inconsistent result of elevated erythrocyte SOD activity in people with CF is due to haemoglobin abnormalities and/or iron supplementation (data regarding nutritional intake/supplementation was not reported) [64]. It is important to note, however, that no correlations were observed between erythrocyte SOD activity and a biomarker of lipid peroxidation in children with CF [85]. Therefore, given these results, it remains unclear whether circulatory SOD activity has a role in the pathophysiology of CF.

Other oxidoreductases included in the present review's meta-analyses were erythrocyte CAT activity and plasma/serum ceruloplasmin content. Notably, there were no differences in erythrocyte CAT activity [53,61] or plasma/serum ceruloplasmin content [70,71] in people with clinically-stable CF compared to controls. However, both of these biomarkers were studied by only 2 trials that were of moderate-to-high quality, but contained modest sample sizes. Furthermore, trials have documented normal plasma and whole blood GPx activity [52,58,85], lowered plasma diamine oxidase activity [71], elevated serum GSTα [88], elevated serum GSTα subunit 1 (GSTα_1_) content [89], elevated erythrocyte glutathione reductase (GR) activity [64], and normal serum γ-glutamyl transferase (GGT) content [46]. However, these biomarkers were not the subject of meta-analysis either due to being studied by only a single trial or by two or more trials from the same laboratory. Further research is required to understand whether, or not, circulating oxidoreductases are suitable therapeutic targets.

### 4.4 Lipid peroxidation

A vulnerability to lipid peroxidation has been associated with reduced longevity of life in non-CF populations [90]. Despite the suggested clinical importance of lipid peroxidation in the pathophysiology of various chronic diseases, the poor specificity of biomarkers is often described as a limiting factor in the study of oxidative stress [91]. However, the emergence of 8-iso-PGF_2α_, a lipid peroxidation product of arachidonic acid, as a biomarker of oxidative stress has vastly improved the progression of the field [92].

Despite a wealth of research investigating 8-iso-PGF_2α_ levels in human disease [22], the present review revealed only 2 independent studies eligible for meta-analysis [58,75], both of which were of high quality (Table 2). Whilst total 8-iso-PGF_2α_ was significantly increased in people with CF versus controls (Fig. 3), neither study reported concentrations of free 8-iso-PGF_2α_, which would allow the conclusion of increased lipid peroxidation as opposed to the simultaneous activity of the inflammatory-induced prostaglandin-endoperoxide synthases [93]. This is important, as a recent meta-analysis summarizing the levels of total and free 8-iso-PGF_2α_ in human disease demonstrated a greater response of free 8-iso-PGF_2α_ in CF (including those who are classified as unstable) versus controls, suggesting that the increased concentrations of 8-iso-PGF_2α_ may be a result of non-specific production [22].

The most studied biomarker of lipid peroxidation in CF was MDA in plasma or serum, which was the topic of 8 trials eligible for meta-analysis [7,54,56,58,61,64,72,74]. Meta-analysis observed significantly elevated concentrations of MDA in the plasma or serum of people with CF versus controls, albeit with a large degree of heterogeneity (Fig. 3). Notably, 5/8 of the trials measured MDA using thiobarbituric acid based assays [56,58,64,72,85]; however, the use of these assays in complex biological samples is cautioned against as many compounds within blood react with thiobarbituric acid to produce coloured adducts that could be mistaken for elevated lipid peroxidation [94]. In contrast, the present review demonstrated that concentrations of lipid hydroperoxides in the plasma or serum of people with CF versus controls were not different; however, meta-analysis was only conducted on 2 trials and a large degree of heterogeneity limited the results (Fig. 3).

Interestingly, lipid peroxidation biomarkers were positively correlated to blood monocyte, neutrophil and white cell counts (Table 3). These correlations suggest that elevated total 8-iso-PGF_2α_ is associated with exacerbated immune stresses and inflammation that characterises CF-lung disease. Indeed, chronic bacterial colonisation of the CF airway leads to a dysregulated cycle of inflammation and redox dysregulation [95], which subsequently leads to cases of oxidative stress [53]. There was not sufficient data to conduct subgroup analysis of 8-iso-PGF_2α_ content in participants with varying degrees of lung disease. However, it must be noted that there was no significant correlations between total 8-iso-PGF_2α_ and FEV_1_ in people with mild-to-moderate CF lung disease [41,75]. Importantly, authors state that whilst spirometry assesses previous lung damage from infection, transient increases in 8-iso-PGF_2α_ may reflect the current pathophysiological state of the lung [96]. Therefore, future trials investigating longitudinal changes in blood 8-iso-PGF_2α_ alongside spirometry may be of interest to determine whether this biomarker is a suitable target for redox-based therapeutics.

Various non-pulmonary consequences of CF have also been suggested to contribute to lipid peroxidation, such as CF-related dysglycaemia [11]. Specifically, exocrine pancreatic insufficiency and variable insulin resistance lead to the development of CFRD in ~35% of adults with CF [4], which is associated with greater declines in lung function [97], nutritional status [98], exercise capacity [99] and prognosis [100]. It was not possible to conduct sub-group analysis on lipid peroxidation biomarkers in people along the dysglycaemic spectrum of CF, and no correlations between lipid peroxidation and glycaemic control were reported by included studies. No studies to date have investigated the content of 8-iso-PGF_2α_ in CFRD. Instead, Ntimbane et al. [11] described significantly increased concentrations (+109%) of 4-hydroxynonenal (HNE)-protein adducts in people with CFRD vs. their CF-counterparts with normal glucose tolerance. Oxidative stress, *in vitro*, is also implicated in pancreatic β-cell dysregulation, and thus, reduced insulin secretion [101]. Therefore, whilst defective CFTR contributes to oxidative stress in CF, it is likely that the dysregulation of various inflammatory-redox cycles also exacerbates cellular damage and contributes to a worsened phenotype (e.g. the progression from impaired glucose tolerance to CFRD) [101].

Whilst the present meta-analysis does provide preliminary evidence that lipid peroxidation is elevated in the blood of clinically-stable people with CF, further high-quality research is required to improve the clarity of our understanding. Prospective observational trials should quantify free and total 8-iso-PGF_2α_ in CF versus healthy control participants. Such data would further the rationale to investigate redox-based therapeutics in CF. In other chronic health conditions, non-pharmaceutical interventions to inhibit lipid peroxidation include dietary and lifestyle modifications (e.g. physical activity promotion), and supplementation of various extracts, oils and juices (e.g. green tea extract); however, such strategies are yet to be investigated in people with CF [102].

### 4.5 Protein carbonylation

Oxidative damage of proteins has been linked with the pathophysiology of many diseases and is most commonly quantified by the determination of protein carbonyls [6]. A number of radical species, including hydrogen peroxide and peroxynitrous acid, readily oxidize amino acid residues to form carbonyl groups. The present review observed significantly elevated concentrations of protein carbonyls in the plasma or serum of people with CF versus controls (Fig. 3). However, included studies did not report correlation coefficients between protein carbonyls and clinically relevant outcomes. In other chronic health conditions, oxidative damage to proteins is a significant contributor to skeletal muscle dysfunction [103], with reports showing elevated concentrations of protein carbonylation and nitration in the blood and skeletal muscle of people with chronic obstructive pulmonary disease [104]. Such associations could have implications in the skeletal muscle dysfunction which characterise CF [105].

It has been suggested that peripheral skeletal muscle dysfunction in CF may be a consequence of reduced skeletal muscle mass due to nutrient malabsorption, physical inactivity and inflammation, but also a defect in skeletal muscle oxidative metabolism [106,107]. More recently, redox disturbances have been suggested to contribute to exercise intolerance in CF [108]. Tucker et al. [108] used electron paramagnetic resonance spectroscopy to determine alkoxyl radical formation during a single bout of submaximal cycling in adolescents with CF (FEV_1_ 93 ± 16% predicted) versus age- and sex-matched healthy controls. It was discovered that whilst circulating protein carbonyls and total 8-iso-PGF_2α_ were not significantly different at baseline or during exercise (notably, in a small sample of adolescents with only mild CF lung disease), there was a significant increase in alkoxyl radical production during exercise in the CF group only [108]. However, the sources for an exacerbated production of reactive oxygen species during exercise in people with CF are unknown.

Defective CFTR has been associated with mitochondrial defects [109], whereby several studies have reported a reduced activity of the mitochondrial complex I in CF cells [[110], [111], [112]], potentially due to mitochondrial GSH depletion [113], and complex V in CF mice (F508del) [114]. However, a marker of mitochondrial oxidative stress, aconitase, was not different in skeletal muscle subsarcolemmal or interfibrillar mitochondria between CF and control mice [114]. Other key non-mitochondrial sources of reactive oxygen species that could contribute to the oxidative modification of protein during exercise are NADPH oxidase, xanthine oxidase and phospholipase A_2_ [115]. However, these enzyme complexes are yet to be studied in models of CF skeletal muscle. It is, therefore, not clear whether redox imbalances are a suitable therapeutic target to improve mitochondrial function and/or skeletal muscle oxidative capacity in CF. This represents an important avenue for future research.

Whilst the present meta-analysis does provide preliminary evidence that protein carbonylation is elevated in the blood of clinically-stable people with CF, further research is required to improve the clarity of our understanding. Prospective observational trials should identify relationships between protein carbonylation biomarkers and clinically relevant outcomes.

### 4.6 Limitations

The present systematic review and meta-analysis had several inherent limitations. At present, there are significant gaps in the literature investigating whether age, sex and genotype affect redox biomarkers. From the correlations reported by the studies included in this review, it appears that antioxidants have a negative, but oxidative stress biomarkers have a positive relationship with age. However, longitudinal studies are required to confirm these associations. A limiting factor of the present review was a high degree of heterogeneity in the majority of biomarkers, which might be explained by only 10/49 studies having matched cases and controls by age and one other factor (Table 2). We, therefore, recommend that future trials must match controls and cases by at least age and, preferably, sex. Other sources of heterogeneity include variations in the methodology of the assays and units of measure.

Another important limitation is the age of some of the included studies. Due to advances in medicine, the survival age of people with CF has substantially increased since the oldest included study in 1977. Furthermore, ground breaking treatments, such as CFTR modulators that alter the negative consequences of CFTR mutations at a molecular level, are becoming mainstream treatment options. It is unknown whether the redox imbalances observed in the present review would persist following CFTR modulator treatment, and this is also an important area for future research.

### 4.7 Future research directions

The present review focussed upon antioxidant and oxidative stress biomarkers that were sampled whilst the cases with CF had clinically-stable lung disease (i.e. not hospitalised). In addition to increased lung infection and inflammation [116], CF-related pulmonary exacerbations are also associated with the development of acute dysglycaemia even in cases with normal glucose tolerance [117], increased muscle weakness and weight loss in bedbound patients [118,119], and hypovitaminosis of antioxidant micronutrients [28] - all of which may increase biomarkers of oxidative damage [6]. Furthermore, whilst the treatment of pulmonary exacerbations may lead to increased antioxidant vitamin content of the blood plasma [120], evidence of elevated lipid peroxidation and protein carbonylation persist despite significant improvements in lung function [120,121]. Therefore, it would be of interest for future trials to investigate whether such redox imbalances following the treatment of acute pulmonary exacerbations are associated with clinically relevant outcomes, such as time until the next hospitalization.

Various biomarkers which are important to understand oxidative stress *in vivo* have not yet been studied on a case-control basis in the blood of people with clinically-stable CF. DNA oxidation can be quantified by circulating 8-OHdG. Systematic evidence suggests blood 8-OHdG is elevated in cardiovascular disease [122]. Furthermore, elevated blood 8-OHdG is associated with poorer prognosis in people with metabolic [123], cardiovascular [124,125] and neurodegenerative [126] diseases. Elevated urinary 8-OHdG has been documented in adolescents with mild to severe CF lung disease versus age-matched controls [127]. Additionally, urinary 8-OHdG was positively associated with plasma α-tocopherol, but a significant correlation was not observed with FEV_1_ [127]. However, various co-morbidities of CF, such as renal disease, are becoming increasingly common [29]. These co-morbidities may cause local inflammation that could reduce the ability of urinary samples to provide systemic indices of oxidative stress. Therefore, it would be useful to confirm these findings in blood samples.

Another increasingly recognised co-morbidity of CF is micro- [128] and macrovascular [129] endothelial dysfunction [130], which may be accentuated by the development of dysglycaemia [131]. Cell culture studies have demonstrated that CFTR is expressed in endothelial cells from the human pulmonary artery [132,133], lung microvasculature [134] and umbilical vein [134], which normally limits, but in CF may exacerbate, the production of pro-inflammatory and pro-oxidant pathways [130]. Previous research has demonstrated that acute treatment of CF participants with antioxidants (1000 mg vitamin C, 600 IU vitamin E, and 600 mg α-lipoic acid) [135] or tetrahydrobiopterin (20 mg/kg) [136] improved endothelial function measured by flow mediated dilation. These data implicate both oxidative stress and a reduced bioavailability of the potent vasodilator, nitric oxide, in CF-related endothelial dysfunction. Notably, in circumstances of oxidative stress, superoxide readily oxidises nitric oxide to form peroxynitrite. Therefore, it would be useful for future studies to quantify levels of nitrotyrosine (a biomarker of nitrosative stress by reactive nitrogen species, including peroxynitrite) in the blood of clinically-stable people with CF versus controls. Notably, previous trials have reported elevated concentrations of nitrotyrosine in the exhaled breath condensate [137] and sputum [138] of people with CF.

## 5 Conclusion

This review is the first to systematically demonstrate that some, but not all, circulating biomarkers of antioxidant capacity are lowered and oxidative stress are increased in people with clinically-stable CF compared to controls. Further research is required to understand the consequences of oxidative stress upon the pathophysiology of CF-related disorders, such as lung disease, diabetes mellitus and skeletal muscle abnormalities.

## Funding

This research was funded by the 10.13039/100009153University of Portsmouth.

## Declaration of competing interest

The authors have no conflicts of interest to declare.
